# State of the art of PET/MRI for rectal cancer assessment

**DOI:** 10.1590/0100-3984.2025.58.e8-en

**Published:** 2025-12-08

**Authors:** Caroline Lorenzoni Almeida Ghezzi, Aline Spader Casagrande, Tiago Leal Ghezzi

**Affiliations:** 1 Hospital Moinhos de Vento, Porto Alegre, RS, Brazil; 2 Hospital de Clínicas de Porto Alegre (HCPA), Porto Alegre, RS, Brazil

Rectal cancer presents significant diagnostic and therapeutic challenges, especially for locally advanced tumors. Accurate tumor-node-metastasis staging is essential for individualized planning, guiding the choice of primary treatment, as well as between the curative or palliative intent of surgery and the indication for additional therapies, given that understaging or overstaging can have a significant impact on patient management and prognosis.

Imaging plays a key role in the preoperative evaluation, allowing assessment of the primary tumor, regional lymph node disease, and detection of distant metastases. According to the latest National Comprehensive Cancer Network (NCCN) guideline^(1)^, preoperative imaging in cases of rectal cancer includes magnetic resonance imaging (MRI) of the pelvis, MRI or computed tomography (CT) of the upper abdomen, and CT of the chest.

Pelvic MRI is considered the main imaging modality in therapeutic planning and prognostic evaluation for patients with rectal cancer, allowing the assessment of the depth of tumor invasion, as well as of the involvement of the mesorectal and lateral pelvic lymph nodes. The examination also provides precise anatomical images of the pelvic structures, including the mesorectal fascia, predicting involvement of the circumferential resection margin, extramural venous invasion, and mesorectal tumor deposits. Therefore, MRI provides information that is essential for individualized decision-making.

It has been shown that MRI has high accuracy in predicting the initial staging of rectal cancer^(2)^, and MRI protocols have been standardized in accordance with the consensus statement issued by the European Society of Gastrointestinal and Abdominal Radiology^(3)^. The use of pelvic MRI for assessment of the response to neoadjuvant treatment and restaging has also been validated in the updated version of that consensus and is necessary for treatment planning^(4)^. Using advanced techniques, pelvic MRI has the ability to assess cellularity through diffusion-weighted imaging (DWI) and tissue perfusion through dynamic contrast-enhanced (DCE) studies, which has also made it the examination of choice according to the NCCN guideline for this scenario.

In addition to the investigation of local (pelvic) involvement in rectal cancer, the evaluation of distant foci is essential, given that, in certain cases, surgical treatment may be indicated even in the presence of metastases. The most common sites of hematogenous spread in colorectal cancer are the liver and lungs. Lung metastases occur in approximately 4-9% of patients with colorectal cancer^(5)^, whereas synchronous liver metastases occur in approximately 20-34%^(6)^.

According to the latest NCCN consensus^(1)^, fluorodeoxyglucose (FDG) positron-emission tomography/CT (PET/CT) is indicated only in specific cases, such as those requiring investigation of residual disease not detected by conventional methods, characterization of indeterminate lesions, evaluation of potentially curable metastatic disease, and investigation of extrahepatic metastatic disease in patients with known liver metastases. It can also be indicated for the staging of patients at high risk of metastases, such as those with extramural venous invasion or elevated serum carcinoembryonic antigen.

The article “State of the art of PET/MRI for rectal cancer assessment: the added value to conventional imaging”^(7)^, published in Radiologia Brasileira, addresses a developing modality, not yet included in current guidelines, constituting a hybrid imaging technique. The technique combines the metabolic assessment of PET with the superior anatomical resolution of MRI, allowing simultaneous analysis of tumor morphology and metabolic activity, thus reducing the total examination time because of the simultaneous acquisition, as well as exposing patients to less radiation in comparison with PET/CT and CT.

Despite the promising potential of PET/MRI in staging and in assessing the treatment response in rectal cancer, its clinical implementation is still limited by the lack of standardized protocols, low availability, and the paucity of validation studies. Variability in MRI sequences, PET acquisition parameters, contrast use, and functional techniques (such as DWI and DCE studies) hinder comparison of results across studies and the practical application of the technique. In addition, the sensitivity of PET/MRI for detecting lung nodules smaller than 5 mm is lower than is that of chest CT and PET/CT, limiting its ability to perform a complete assessment of metastatic disease^(8)^.

There is a need for multicenter prospective studies to determine the optimal acquisition and reconstruction parameters for PET/MRI in rectal cancer, to validate metabolic and functional biomarkers that can predict treatment response and prognosis, and to directly compare PET/MRI with conventional methods (pelvic MRI, thoracic/abdominal CT, and PET/CT) to define the precise clinical indications.

To allow the future integration of PET/MRI into clinical guidelines, it is essential to standardize the protocols, making it a robust instrument for individualized planning and therapeutic decision-making in rectal cancer.

The future prospects for PET/MRI in rectal cancer are promising. Combining PET/MRI with advanced functional sequences (DWI and DCE studies) allows simultaneous assessment of tumor morphology and metabolism, thus optimizing surgical planning and enabling adaptive adjustments to the treatment plan.

In FDG PET imaging, the limitations of the standardized uptake value-used as a semiquantitative parameter to measure the radiotracer concentration in the tissue and to identify neoplastic tissue-can be overcome by using FDG PET/MRI to measure the volumetric parameters, such as the metabolic tumor volume and total lesion glycolysis, which reflect the overall metabolic activity of the tumor mass^(9)^.

The future of PET/MRI lies in the integration of radiomics, artificial intelligence, and new radiotracers, such as the fibroblast activation protein inhibitor (FAPI) used in PET. Radiomics allows the extraction of complex quantitative features from images, whereas artificial intelligence correlates those data with clinical outcomes, enabling risk stratification, prediction of the treatment response, and personalized therapeutic decision-making. In addition to FDG, recent developments in PET/MRI include techniques that use other radiotracers, such as FAPI PET, which targets activated fibroblasts in the tumor matrix. Colorectal cancer exhibits high FAP expression, a characteristic that favors the application of FAPI as a radiotracer. The use of FAPI PET allows visualization of the tumor stroma, providing information complementary to FDG-based metabolic imaging, increasing sensitivity for the detection of primary lesions, local invasion, and metastases. In addition, FAPI PET offers new biomarkers of a response to neoadjuvant therapy and potentially identifies areas of persistent stromal activity, which are associated with a higher risk of recurrence^(10)^.
